# Inhibition of IGF-1 Signalling Enhances the Apoptotic Effect of AS602868, an IKK2 Inhibitor, in Multiple Myeloma Cell Lines

**DOI:** 10.1371/journal.pone.0022641

**Published:** 2011-07-25

**Authors:** Ines Tagoug, Amélie Sauty De Chalon, Charles Dumontet

**Affiliations:** 1 Université de Lyon, Lyon, France; 2 INSERM U1052, Centre de Recherche de Cancérologie de Lyon, Lyon, France; 3 CNRS UMR 5286, Centre de Recherche de Cancérologie de Lyon, Lyon, France; 4 HCL, Lyon, France; University Health Network, Canada

## Abstract

Multiple myeloma (MM) is a B cell neoplasm characterized by bone marrow infiltration with malignant plasma cells. IGF-1 signalling has been explored as a therapeutic target in this disease. We analyzed the effect of the IKK2 inhibitor AS602868, in combination with a monoclonal antibody targeting IGF-1 receptor (anti-IGF-1R) in human MM cell lines. We found that anti-IGF-1R potentiated the apoptotic effect of AS602868 in LP1 and RPMI8226 MM cell lines which express high levels of IGF-1R. Anti-IGF-1R enhanced the inhibitory effect of AS602868 on NF-κB pathway signalling and potentiated the disruption of mitochondrial membrane potential caused by AS602868. These results support the role of IGF-1 signalling in MM and suggest that inhibition of this pathway could sensitize MM cells to NF-κB inhibitors.

## Introduction

Multiple myeloma is characterized by unrestrained accumulation of antibody-secreting plasma cells in the bone marrow, attributed to loss of apoptotic control and cell cycle deregulation [1], [2]. Its incidence is approximately 4/100,000 persons per year, but is predicted to increase in the future due to the expected increase in longevity. The proliferation and the survival of MM cell lines and fresh human cells has been shown to be related to the activation of several pathways such as phosphatidylinositol-3 kinase (PI-3K)/Akt, Janus kinase (JAK)/signal transducer and activator of transduction 3 (STAT3), mitogen-activated protein kinase (MAPK)/extracellular signal-regulated kinase (ERK) and nuclear factor kappa-B (NF-κB) [3], [4], [5], [6], [7]. Several growth factors produced by the microenvironment induce the activation of these pathways such as interleukin 6 (IL-6), and insulin-like growth factor 1(IGF-1) [8], [9]. *In vivo* and *in vitro*, IGF-1 decreases drug sensitivity of MM cells, and upregulates a series of anti-apoptotic proteins such as A1/Bfl-1, XIAP and Bcl-2 [10], [11], [12].

Several strategies seek to target and inhibit the IGF-1 signalisation pathway, either by blocking the IGF-1 receptor or by inhibiting the signals downstream of this receptor [13], [14], [15]. Monoclonal antibodies targeting the IGF-1R constitute a very attractive approach to block the IGF-1/IGF-1R interaction, subsequently blocking the stimulation of several signalisation pathways, especially PI-3K/Akt and NF-κB [16]. Inactive NF-κB is located in the cytosol associated to different NF-κB inhibitory proteins (IκB) [17]. Many stimuli such as growth factors, stress and inflammatory response induce the phosphorylation of IκB proteins, thereby activating their ubiquitination and degradation by the 26S proteasome [18]. The IκB kinase complex (IKK) is responsible for the phosphorylation of IκB. IKK include three subunits, the two catalytic subunits IKK1 (IKKα) and IKK2 (IKKβ), and the regulatory subunit Nemo (IKKγ) [19], [20], [21]. The IKK kinase complex indirectly regulates the translocation of NF-κB to the nucleus and the transcription of growth and survival genes. While inhibition of the NF-κB pathway has been suggested to be a significant mechanism of proteasome inhibitors such as bortezomib [22], another approach to inhibit this pathway consists in the inhibition of IKK [23]. IKK inhibitors could have anticancer effects *per se* or could potentiate agents interfering on signalisation pathways upstream of NF-κB, as seems to be the case for IGF-1R inhibitors.

AS602868 is an anilinopyrimide derivative and adenosine triphosphatase competitor selected for its inhibitory effect on IKK2 *in vitro*. AS602868 induces the apoptosis of MM, acute myeloid leukemia and sarcoma cell lines *in vitro* [24], [25], [26]. It has also been shown that AS602868 blocks the canonical NF-κB pathway and the proliferation of MM cell lines [27].

Recent work from our laboratory recently identified gene expression profiles in fresh human acute myeloid leukemia cells exposed to AS602868 and suggests that multiple mechanisms, including pathways other than NF-κB may be induced by this agent [28].

In this study, we analysed the effect of the combination of the IKK2 inhibitor AS602868 and a monoclonal antibody directed against IGF-1R on MM cell lines. AS602868 was found to block the activation of NF-κB, induced a dissipation of mitochondrial transmembrane potential and induced apoptosis in MM cell lines. The combination of the anti IGF-1R antibody with AS602868 increased the cytotoxic effect in MM cell lines, suggesting that simultaneous targeting of IGF-1 signalling and the NF-κB pathway could be of therapeutic value in multiple myeloma.

## Results

### IGF-1R expression level

**Figure 1:**
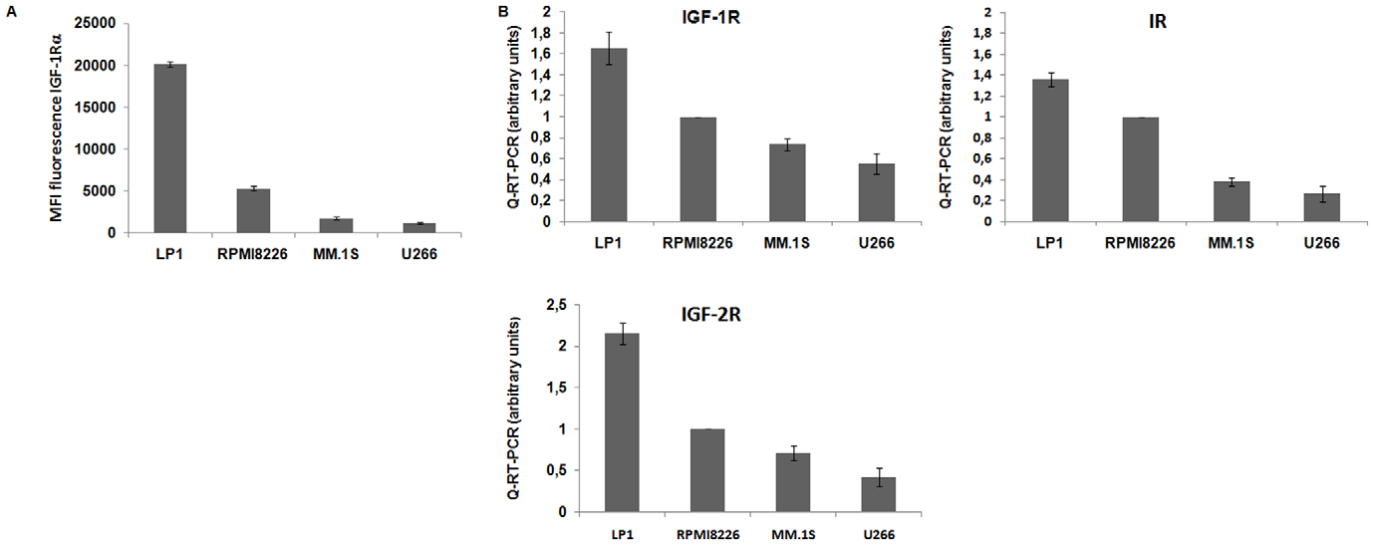
Level expression of IGFs receptors on four MM cell lines. 1.A Level expression of IGF-1Rα by flow ctometry on RPMI8226, LP1, MM.1S and U266. 1.B Expression of IGF-1R, IR and IGF-2R by RT-PCR on RPMI8226, LP1, MM.1S and U266.

In a first step, we measured the expression level of IGF-1Rα by flow cytometry in the different MM cell lines. We found that RPMI8226 and LP1 expressed more IGF-1Rα than MM.1S and U266 (Figure 1A). Since IGF-1 can also bind the insulin receptor (IR) and the insulin-like growth factor 2 receptor (IGF-2R), we analyzed the expression of IGF-1R, IGF- 2R and IR by quantitative RT-PCR. We confirmed that LP1 and RPMI8226 expressed a higher level of IGF receptor than MM.1S and U266 (Figure 1B).

### Combining the anti-IGF-1R monoclonal antibody with the IKK2 inhibitor AS602868 increases the apoptosis of MM cell lines

**Figure 2:**
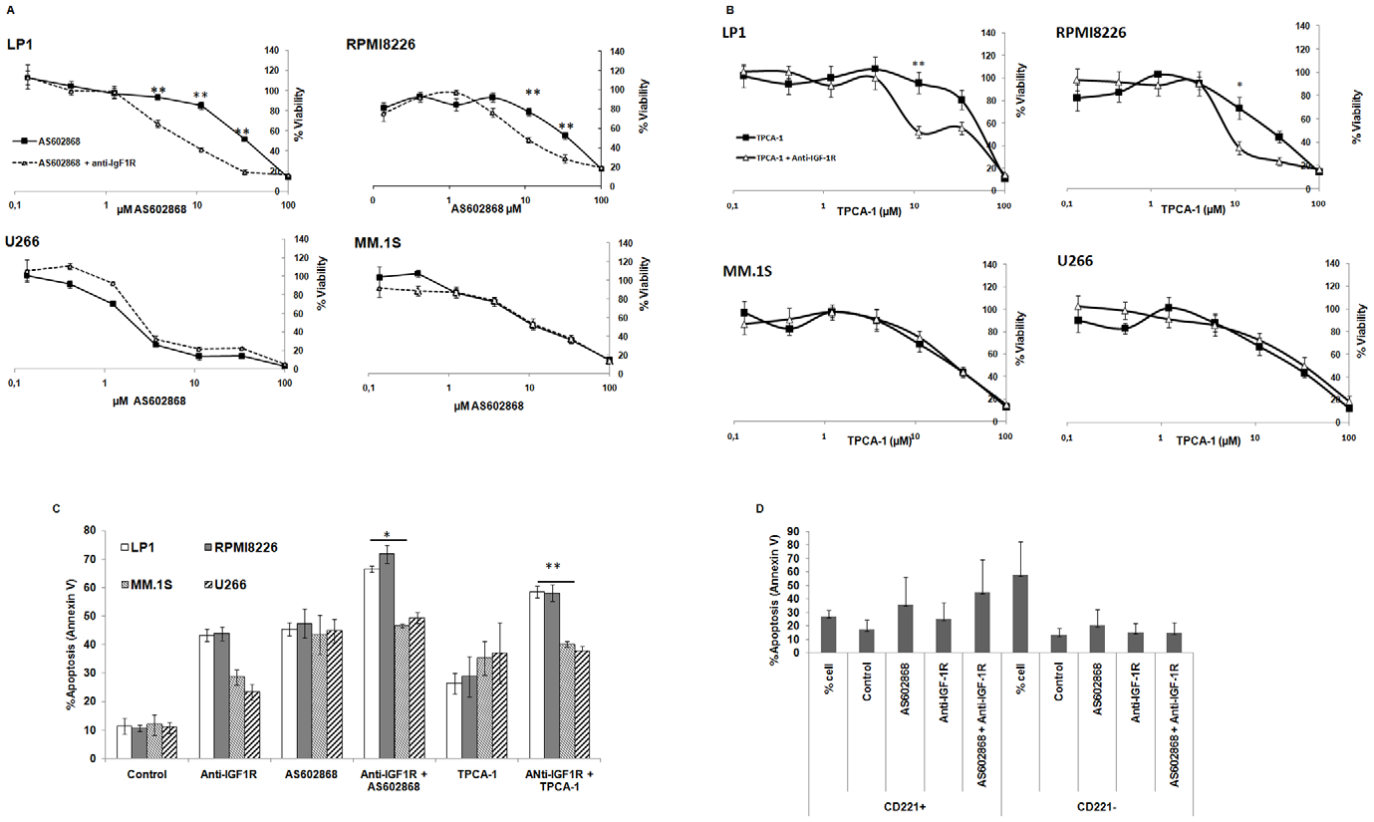
Anti-IGF-1 antibody enhances the cytotoxic effect of IKK2 inhibitors on RPMI8226 et LP1. 2.A Anti-IGF-1R antibody enhanced the cytotoxic effect of AS602868 on RPMI8226 and LP1 cell lines. Cells were plated in triplicate and exposed to various concentrations of AS602868 in the presence or absence of anti-IGF-1R antibody at a fixed concentration of 10 µg/mL. Cytotoxicity was evaluated using an MTT assay. Cells exposed to AS602868 alone were compared to controls cultured in serum alone. Cells exposed to AS602868 in the presence of Anti-IGF-1R antibody were compared to controls cultured in presence of Anti- IGF-1R antibody alone. 2.B Anti-IGF-1R antibody enhanced the cytotoxic effect of TPCA-1 on RPMI8226 and LP1 cell lines. Cells were plated in triplicate and exposed to various concentrations of TPCA-1 in the presence or absence of anti-IGF-1R antibody at a fixed concentration of 10 µg/mL. Cells exposed to TPCA-1 alone were compared to controls cultured in serum alone. Cells exposed to TPCA-1 in the presence of Anti-IGF-1R antibody were compared to controls cultured in presence of Anti-IGF-1R antibody alone. 2.C Anti-IGF-1 R potentiated the apoptotic effect of IKK2 inhibitors AS602868 and TPCA-1 on RPMI8226 and LP1 MM cell lines. Cells were exposed for 72 h with 10 µM AS602868 or 10 µM TPCA-1 in the presence or absence of anti-IGF-1R antibody at a fixed concentration of 10 µg/mL then analyzed by flow cytometry after annexin V staining. Mean± SD of three experiments ** *P*<0.001: * *P*<0.05 versus control. 2.D Effect of anti-IGF-1R antibody+AS602868 on fresh human myeloma samples according to CD221 (IGF-1Rα) status. This combination induced more apoptosis on CD221hi plasma cells than in CD221lo cells. Cells were incubated with 10 µM AS602868 or/and anti-IGF-1R antibody for 24 h. Apoptosis was analyzed by flow cytometry.

In order to study the effect of the combination of the IKK2 inhibitor AS602868 and the anti- IGF-1R monoclonal antibody on four human MM cell lines, we incubated cells with various concentrations of AS602868 alone or in combination with 10 µg/mL anti-IGF-1R. While AS602868 was cytotoxic against the four cell lines studied, the combination with anti-IGF-1R antibody enhanced cytotoxicity significantly only in RPMI8226 and LP1 cells (Figure 2A). IC50 for AS602868 were reduced from 35 µM to 10 µM upon addition of anti-IGF-1R in RPMI8226 cell line and 39 µM to 7 µM in LP1 cell line. The combination of anti-IGF-1R with AS602868 had not any additional effect on MM.1S and U266 cell lines (Figure 2A) which express lower levels of IGF-1R. Same experience is performed without serum; such as the first experience, the effect effect of AS602868 was enhanced by anti-IGF-1R antibody (Figure S2).

To determine if the potential effect of anti-IGF-1R was related to the inhibition of IKK2, we studied the viability and apoptosis of MM cell lines in presence of the selective IKK2 inhibitor, TPCA-1. This inhibitor decreased the viability of the four cell lines. However, the cytotoxic effect of TPCA-1 was enhanced by anti-IGF-1R only in LP1 and RPMI8226 cells (Figure 2B).

To confirm these results, we measured the induction of apoptosis by annexin V staining in cells exposed to 10 µM AS602868, TPCA or/and 10 µg/mL anti-IGF-1R antibody. Interestingly, the four cell lines showed an apoptotic response upon incubation for 72 hours with 10 µM AS602868, TPCA-1 or/and 10 µg/mL anti-IGF-1R. However, anti-IGF-1R antibody enhanced apoptosis induced by these agents only in the in LP1 and RPMI8226 cell lines (Figure 2C). Growth inhibition studies showed that MM.1S and U266 were more resistant to anti-IGF-1 then LP1 and RPMI8226 (Figure S1). The combination of anti-IGF- 1R with AS602868 or with TPCA-1 was found to increase the apoptotic fraction in RPMI8226 and LP1 cells but not in MM.1S and U266 cells (Figure 2C). To understand how the inhibition of IKK2 and the IGF-1R antibody could cooperate to cause cell death in myeloma cells we then chose to focus on the two cells lines with the highest levels of IGF-1R, LP1 and RPMI8226.

### Combining the anti-IGF-1R monoclonal antibody with the IKK2 inhibitor AS602868 increases the apoptosis of primary myeloma cells

Since anti-IGF-1R antibody enhanced the cytotoxicity effect of the IKK2 inhibitors on MM cell lines, we evaluated the effect of this combination on primary MM cells. We incubated primary myeloma cells with IKK2 inhibitors or/and anti-IGF-1R antibody for 24 h. We analyzed the effect of these signalling pathways inhibitors on CD221+ and CD221− plasma cells. About 30% of primary plasma cells express IGF-1R (CD221+) (Table S3). After 24 h, anti-IGF- 1R antibody increased the apoptotic fraction from 5% to 20% in CD221+ plasma cells with no significant effect on CD221− plasma cells (Figure 2D). The effect of AS602868 was more pronounced on CD221+ plasma cells then on CD221− plasma cells. The apoptotic effect of the combination was detected only on CD221+ plasma cells (Figure 2D). Anti-IGF-1R antibody and AS602868 were not any cytotoxicity effect on bone marrow non plasma cell compartment (Table S4).

### Effect of AS602868 and anti-IGF-1R antibody on cell cycle progression in MM cell lines

**Figure 3:**
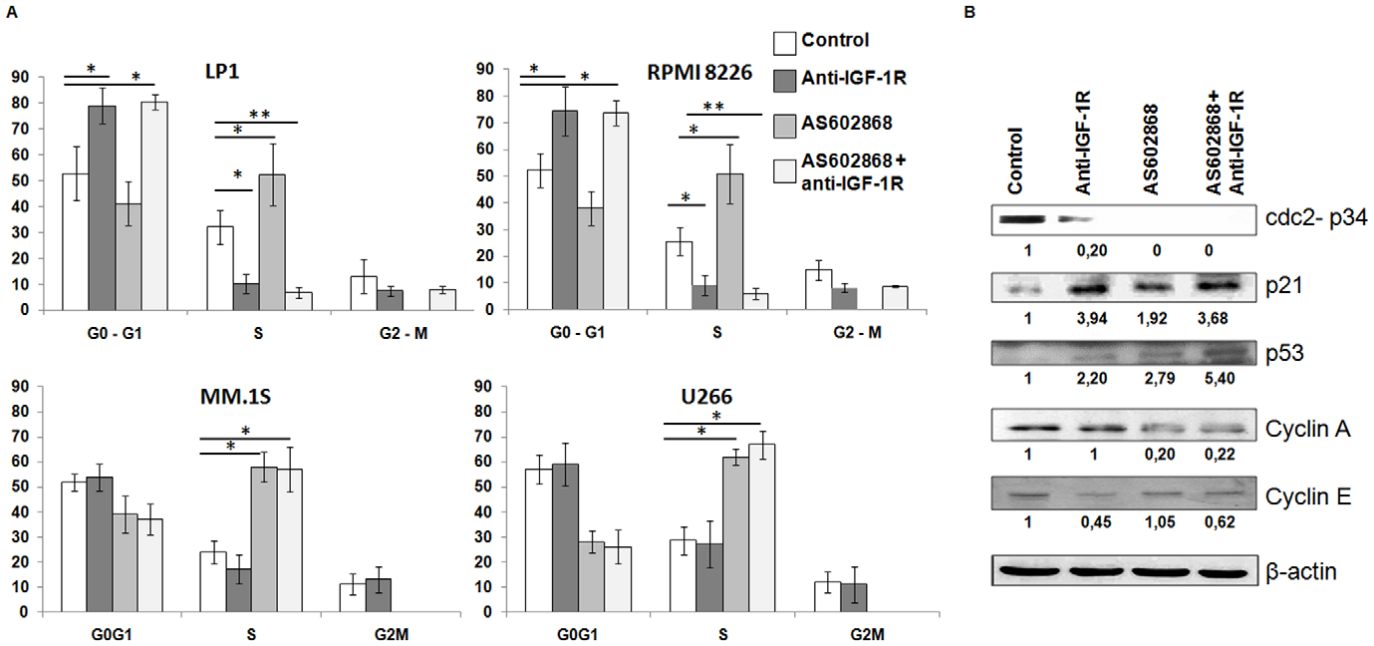
AS602868 and anti-IGF-1R antibody block the cell cycle in RPMI8226 and LP cell lines. 3.A Effect of AS602868 and anti-IGF-1R antibody on the cell cycle. MM cell lines were exposed for 40 h to 10 µM AS602868 or/and 10 µg/mL anti-IGF-1R antibody at 37°C. Cells were incubated with propidium iodide and then analyzed with flow cytometry. 3.B Analysis of cell cycle proteins in RPMI8226 cells exposed to AS602868 or/and anti- IGF-1R. RPMI8226 cells were incubated with 10 µM AS602868 or/and anti-IGF-1R antibody 10 µg/mL for 16 h then examined by Western blotting. Mean± SD of three experiments ** *P*<0.001: * *P*<0.05 versus control.

In order to determine the effect of AS602868 and/or anti-IGF-1R on cell cycle progression, we exposed LP1, RPMI8226, MM.1S and U266 cells lines to 10 µM AS602868 and/or 10 µg/mL anti-IGF-1R antibody. AS602868 alone caused a strong block of cells in the S phase of the cell cycle in the four cell lines, whereas anti-IGF-1R antibody blocked cells in G0–G1 phase only in the LP1 and RPMI8226 lines. When cells were exposed to both molecules, more than 70% of LP1 and RPMI8226 cells were blocked in G0–G1 (Figure 3A). Of note the degree and pattern of cell cycle blockage was quite similar in these two IGF-1R positive lines. Cdc2 p34 is essential for the G2 to M transition. In this study, the analysis of the expression level of cdc2 p34 showed that this protein became undetectable after exposure to AS602868 in the RPMI8226 cells, either alone or in combination with anti-IGF-1R antibody (Figure 3B). We also analysed the level expression of cyclin A. AS602868 decreased the expression of cyclin A. This reduced content is compatible with the S phase block observed in cells exposed to AS602868. We also analysed the expression level of the cyclin D inhibitor, p21. As shown in Figure 3B, anti-IGF-1R alone or in combination with AS602868 enhanced the expression level of p21 and reduced the expression level of cyclin E which is in keeping with the G0/G1 block observed in cells exposed to anti-IGF-1R (Figure 3B). The expression level of the tumor supressor P53 increased in cells incubated with anti-IGF-1R or AS602868, as well as in cells exposed to the combination of anti-IGF-1R and AS602868 (Figure 3B).

### Effect of AS602868 and anti-IGF-1R antibody on mitochondrial membrane potential

**Figure 4:**
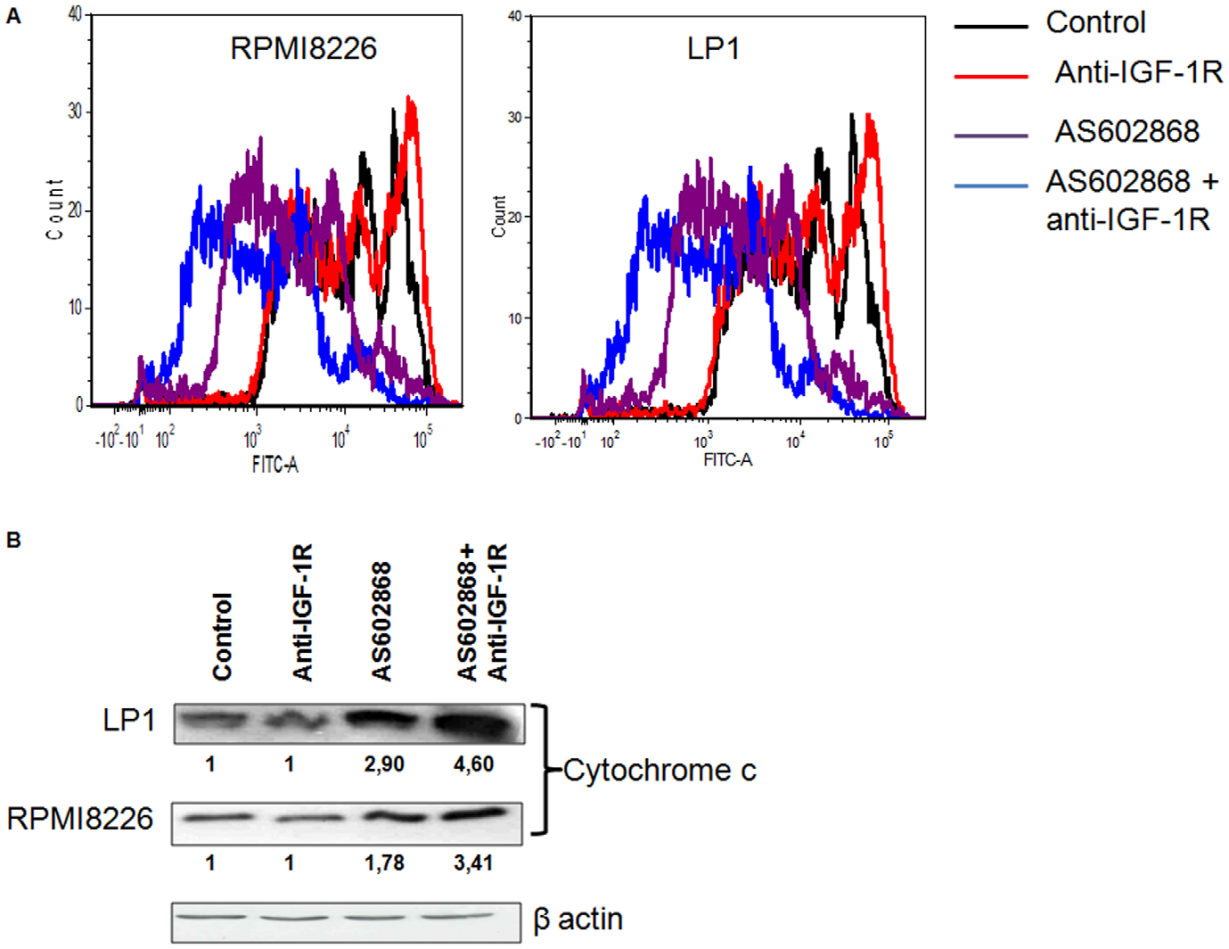
AS602868 induces the depolarization of mitochondrial membrane. 4.A AS602868 and anti-IGF-1R antibody increased the depolarization of mitochondrial membrane in RPMI8226 and LP1 cell lines. Cells were incubated with 10 µM AS602868 or/and anti-IGF-1R antibody 10 µg/mL for 16 h then analysed by flow cytometry. 4.B AS602868 increased the level expression of cytochrome c in RPMI8226 and LP1 cell lines. Cells were incubated with 10 µM AS602868 or/and anti-IGF-1R antibody 10 µg/mL for 16 h.

Apoptosis can be related to the release of pro-apoptotic proteins from mitochondria. In order to determine the apoptotic mechanisms triggered by AS602868 and anti-IGF-1R, we analysed the mitochondrial potential in MM cells by DiOC6 (3). In RPMI8226 and LP1, AS602868 induced depolarization of the mitochondrial membrane (Figure 4A). While anti-IGF-1R in itself did not cause depolarization of mitochondrial membrane its combination with AS602868 caused a greater dissipation of mitochondrial potential than did AS602868 alone. To determine whether these alterations in mitochondrial membrane potential were correlated with apoptotic signalling we studied the expression level of cytochrome c by western blot by flow cytometry in RPMI8226 and LP1 cells. We detected an increase of cytochrome c protein content in cells incubated with AS602868. While anti IGF-1R antibody had not effect on cytochrome c content its association with AS602868 clearly increased the increase of cytochrome c, in particular in LP1 cells which are those with the highest level of expression of IGF-1R (Figure 4B).

### Effect of AS602868 and anti-IGF-1R antibody on NF-κB signalling

**Figure 5:**
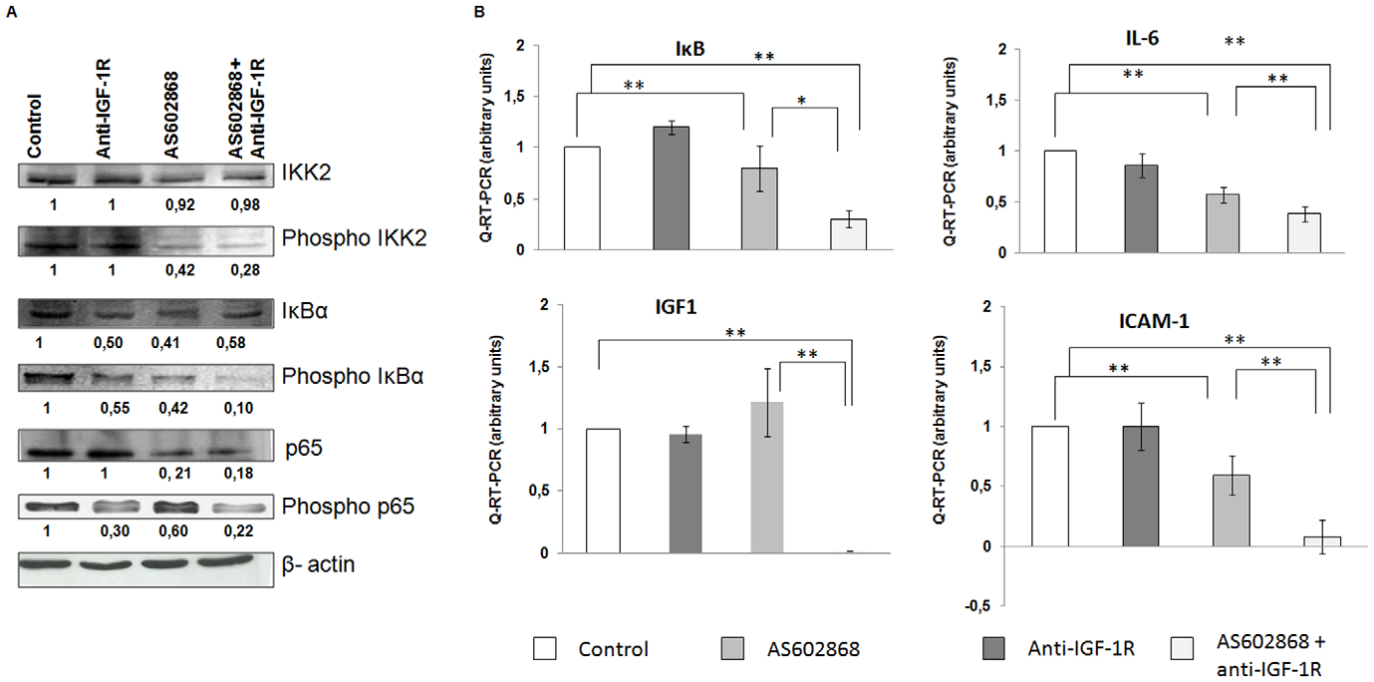
AS602868+anti-IGF-1R antibody inhibit the NF-κB pathway in RPMI8226 and LP1 cells. 5.A AS602868 and anti-IGF-1R antibody decreased the level of NF-κB proteins pathways. RPMI8226 cells were treated for 16 h with 10 µg/mL anti-IGF-1R antibody or/and 10 µM AS602868 at 37°C. 5.B AS602868 and anti-IGF-1R antibody inhibited the expression of NF-κB regulated genes in multiple myeloma (IKB, IGF1, IL-6 and ICAM-1). Mean± SD of three experiments ** *P*<0.001: * *P*<0.05 versus control.

During activation, IKK2 is phosphorylated and induces the phosphorylation of IκBα. In order to study the effect of the combination anti-IGF-1R and the IKK2 inhibitor on NF-κB, we analysed the phosphorylation status of IKK2 as well as that of its substrate IκBα by western blot. As expected incubation of RPMI8226 with AS602868 for 16 h decreased the phosphorylation of IKK2 with a resulting decrease in phospho-IκBα. AS602868 also caused a decrease in p65 but no change in phospho-p65. Conversely anti-IGF-1R antibody decreased the phosphorylation of both IκBα and p65, suggesting that blockage of IGF-1 signalling has a profound inhibitory effect on NF-κB signalling (Figure 5A). Remarkably the reduction of phospho-IκBα was maximal when anti-IGF-1R antibody was combined to AS602868. These results suggest that inhibition of IGF-1 signalling potentiates the repression of NF-κB by an IKK inhibitor such as AS602868.

The ability of the combination of anti-IGF-1R and an IKK2 inhibitor to interfere with NF-κB activation was also analyzed by RT-PCR. Generally, the activation of NF-κB is accompanied with an increased mRNA content of various molecules such as IL-6 and Inter-Cellular Adhesion Molecule 1 (ICAM-1). In RPMI cell lines, we observed that anti-IGF-1R does not have a significant effect on IKB, IGF-1, IL-6 and ICAM-1 (Figure 5B). While the IKK2 inhibitor decreased the expression level of IκB, IL-6 and ICAM-1 (Figure 5B), this effect was more pronounced, both in LP1 and RPMI8226 cells, when anti-IGF-1R antibody was combined with AS602868.

Finally, we studied by RT-PCR the level of IGF-1. Interestingly, we found that the combination suppressed the level of IGF-1 whereas anti-IGF-1R or AS602868 alone did not have any effect on IGF-1 mRNA content in these cells (Figure 5B). Level expression of IGF-1 protein was detected also by western blot. The level expression of protein IGF-1 decreases in cell exposed to the combination anti-IGF-1R and AS602868 (Figure S3).

### Effect of AS602868 and anti-IGF-1R antibody on PI-3K/Akt pathways

**Figure 6:**
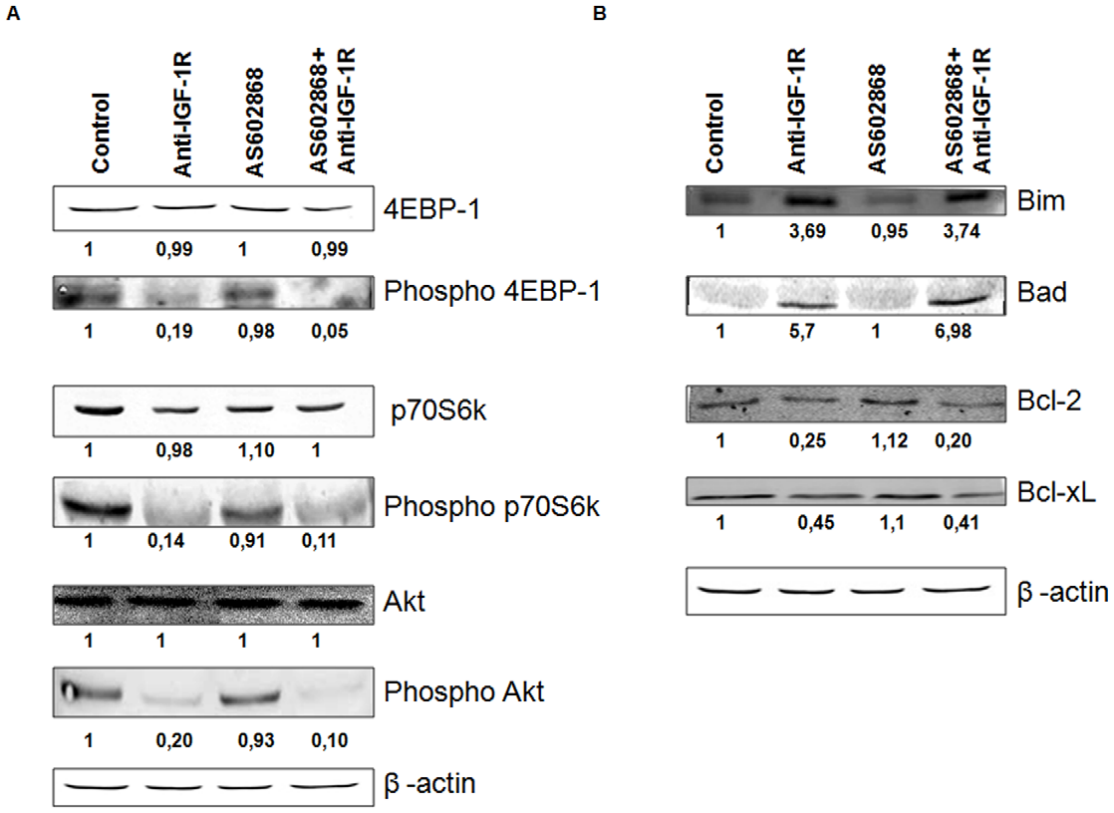
Effect of AS602868 and anti-IGF-1R antibody on PI-3K/Akt pathway and proand anti-apoptotic proteins in RPMI8226 and LP1 cell lines. 6.A Anti-IGF-1R antibody inhibits the phosphorylation of Akt, p70S6K and 4EBP-1 in RPMI8226. 6.B Anti-IGF-1R antibody induces the expression of pro-apoptotic proteins. Cells were incubated for 16 h with 10 µg/mL anti-IGF-1R antibody or/and 10 µM AS602868.

Since IGF-1 is involved in the activation of PI-3K/Akt pathways, we analysed the effect of its inhibition on the RPMI8226 cell line. We observed that the phosphorylation of Akt decreased in cells exposed to anti-IGF-1R. This inhibition of Akt phosphorylation causes a subsequent inhibition of the phosphorylation of the mTor pathway proteins, p70S6K and 4EBP1 (Figure 6A).

### Effect of AS602868 and anti-IGF-1R antibody on Bcl-2 family proteins

As anti-IGF-1R-induced apoptosis of MM cell lines was not associated with depolarization of the mitochondrial membrane, we sought to determine whether alteration in IGF-1 signalling could alter the content of pro-apoptotic and anti-apoptotic proteins. We observed that anti- IGF-1R antibody increased the content of pro-apoptotic proteins Bim and Bad and decreased those of anti-apoptotic proteins Bcl-2 and Bcl-xL (Figure 6B). AS602868 did not affect the content of these proteins under these conditions.

## Discussion

Despite the fact that several treatments for multiple myeloma are currently available, the median survival of multiple myeloma remains in the order of 5 years, in particular in patients who are not eligible for high dose therapy. Many studies proved the importance of the activation of signalling pathways in chemoresistance, cell survival, and proliferation of human multiple cells lines [30]. This makes these signalling pathways a therapeutic target in multiple myeloma. Over the past few years, several studies have shown that inhibition of these signalling pathways by specific inhibitors or by blocking the activator of these pathways causes an inhibition of cell proliferation [31], [32]. The proliferation and the survival of MM cells are strongly dependent on the activation of signalization pathways by cytokines and growth factors [33], [34], [35]. Most of these cytokines and growth factors are secreted by the microenvironment, in particular the bone marrow stromal cells [36], [37]. IGF-1 is now considered as a major growth factor in MM [38]. Previous studies identified a role of IGF-1 as a growth and survival factor in MM. *In vivo* and *in vitro* studies proved IGF-1 increase antiapoptotic proteins (such as Bcl-2, Bcl-XL, cIAP-1, cIAP-2, FLIP) and decrease pro apoptotic proteins (such as caspase 3, caspase 8, caspase 9) and plays a role in drug resistance (dexamethasone, rapamycine)[39], [40], [41] Many studies in multiple myeloma have shown that the role of IGF-1 is correlated with signalling pathway activation. IGF-1 plays a major role in NF-κB, PI-3K/Akt and ras/MaPK activation [12], [30], [42].The inhibition of the interaction between IGF-1 and its receptor is being explored as a therapeutic target in this disease [43], [44], [45], [46]. *In vitro* and *in vivo* studies have proved that the inhibition of of IGF-1R decreased cell proliferation [16], [44], [47]. Our results confirm the wide-ranging effect of IGF-1 inhibition on myeloma cells, including blockage of the G1 to S phase, reduced PI3K signalling and altered equilibrium of pro- and anti-apoptotic proteins. We show that the cytotoxic effect of anti-IGF-1R is more important on MM cell lines with a high level of IGF- 1R. In primary MM cell lines, anti-IGF-1R antibody enhanced the apoptotic effect of the IKK2 inhibitor AS602868 only in plasma cells with high expression of IGF-1R. Constitutive nuclear NF-κB activity has been described in many MM cells lines and primary myeloma cells [48]. Spontaneous and abnormal activation of NF-κB has been related to proliferation and drug resistance of MM cells, confirming the importance of inhibing NF-κB as a therapeutic target in MM [5]. MM cells have been shown to be sensitive to NF-κB inhibitors including proteasome inhibitors and IKK inhibitors [22], [49], [50]. Preclinical and clinical studies have shown that the IKK2 inhibitors AS602868 and TPCA-1 induce apoptosis in MM cells by decreasing the canonical NF-κB pathway [27]. In our study, we observed the effect of the combination of monoclonal anti-IGF-1R antibody and IKK2 inhibitors. Interestingly, among the four cell lines with different expression levels of IGF-1R which we studied, only in those with the highest levels did we observe enhanced cytotoxic activity of IKK2 inhibitors by anti-IGF-1R antibody. We observed that the apoptotic response of MM cells to AS602868 involved disruption of the mitochondrial transmembrane potential and release of cytochrome c. While anti-IGF-1R antibody did not induce mitochondrial membrane depolymerisation it did alter the expression levels of pro- and anti-apoptotic proteins, possibly through inhibition of PI3K signalling, a factor which might explain why the combination with AS602868 resulted in greater cytochrome c content than that caused by AS602868 alone.

Moreover, inhibition of IGF-1 signalling by the anti-IGF-1R antibody, while in itself did not reduce NF-κB signalling, resulted in decreased phospho-IκB, thereby setting the stage for the effect of AS602868 on NF-κB signalling. The inhibition of NF-κB was associated with a reduction in the expression of adhesion molecule ICAM-1 and IL-6. In this study, we show that the combination of two pathways inhibitors decreased the production of IGF-1 and IL-6. Altogether, our data underscore the central role for IGF-1 in MM and the importance of IGF-1 signalling as a potential therapeutic target. Interestingly our results suggest that inhibition of IGF-1 signalling in MM could be used as a chemosensitization strategy rather than as a cytotoxic approach *per se*. We indeed observed that exposure to anti-IGF-1R antibody contributed to mitochondrial membrane disruption and NF-κB inhibition caused by the IKK inhibitor AS602868. These results were all the more remarkable as these two agents had different effects on the cell cycle and that the combination of the two had an effect super imposable to that of the anti-IGF-1R antibody. These findings suggest that a strategy combining monoclonal antibody against IGF-1R with NF-κB inhibitors may constitute a promising therapeutic approach in patients with MM.

## Materials and Methods

### Cell lines

Human multiple myeloma cell lines LP1, RPMI8226, U266 and MM.1S were cultured in RPMI 1640 containing L-Glutamine (Invitrogen, Cergy Pontoise, France), 10% fetal bovine serum (Invitrogen, Cergy Pontoise, France) and 1% penicillin streptomycin (Invitrogen, Cergy Pontoise, France) at 37°C in humidified 95% air and 5% CO2. LP1, MM.1S and U266 were provided by ATCC-LGC (Molsheim, France). RPMI8226 was provided by ECACC from sigma-Aldrich (Sigma Aldrich, St Louis, USA).

### Growth inhibition Assay

The cell survival was assessed by using a 3-(4,5-dimethylthiazol-2-yl)-2,5- diphenyltetrazolium bromide assay (MTT, Sigma Aldrich, St Louis, USA). Cells were cultured in 96-well plates (Costar, NY, USA) for 72 h in the presence of AS602868 (generously provided by Merck-Serono, Geneva, Switzerland), TPCA-1(Sigma Aldrich, St Louis, USA) alone or with an anti-IGF-1Rmonoclonal antibody (generously provided by Roche, Penzberg, Germany). Cells were then incubated with 100 µg MTT, and the resulting crystals were resuspended in 100 µL of isopropanol/0.1 N HCl (Sigma Aldrich, St Louis, USA). Absorbance was measured at 540–690 nm using a spectrophotometer (Thermo Electron Corporation), and inhibitory concentrations 50 (IC50) were determined from cell survival curves drawn with Excel (Microsoft).

### Fresh human myeloma cell studies

We received 2 mL samples of bone marrow (BM) from patients with multiple myeloma, after having obtained written informed consent. Samples were lysed with lysis buffer for 10 minutes at room temperature. BM cells were washed and suspended in 300 µl phosphate buffer (PBS). Cells were cultured in 24 well plates (Costar, NY, USA) at 37°C with AS602868 (10 µM), anti-IGF-1R (10 µg/mL) or AS602868+anti-IGF-1R. After 24 h, cells were washed with PBS for 5 minutes, centrifuged at 600 g at room temperature and incubated with labelled monoclonal antibodies directed against CD 38, CD 138, CD221(IGF-1R alpha) and CD45 (antibodies Reference in Table S2) for 15 minutes in the dark at room temperature. Cells were washed with PBS and suspended in 100 µL Annexin buffer with 1 µl Annexin-V FITC and analysed by flow cytometry on a FACS Canto II (BD Biosciences, Erembodegem, Belgium).

### Western blot analysis

Cells (1.107) were exposed to 10 µM AS602868 or/and 10 µg/mL anti-IGF-1R for 16 hours. Following treatment, cells were washed with phosphate-buffered saline (PBS) and then solubilised for 30 minutes on ice in lysis buffer (20 mmol/L Tris-Hcl (pH 6.8), 1 mmol/L MgCl2, 2 mmol/L EGTA, 0.5% NP40) with the complete mixture of protease inhibitors (leupeptin, aprotinin, benzamidine, PMSF, TRCK). After incubation, cell debris and nuclei were removed by centrifugation at 12,000 g for 15 minutes at 4°C. Equal amounts of total protein (50 µg per lane) combined with Laemmli buffer were boiled at 95°C for 5 minutes, and then separated on 12% sodium dodecyl sulphate (SDS)-polycrylamide gels followed by electrophoretic transfer to iBlot Gel transfer stacks PVDF or nitrocellulose (Invitrogen). Membranes were blocked for 1 hour at room temperature with phosphate-buffered saline containing 0.1% Tween® 20 (PBS-T) and 5% dried milk and incubated for all night at 4°C with specific antibody diluted with PBS-T and 5% non fat milk. The antibodies used were directed against Bcl-2 (M0887, 1/500, Dako, Denmark), Bcl-XL (sc-634,1/500, Santa cruz, USA), IGF-1 (sc-1422, 1/500, Santa cruz, USA), P21(sc-397, 1/500, Santa cruz, USA), P53 (clone DO7, 1/1000, Dako, Denmark), IkBα(ser32/ser36, IMG-156,1/500 IMGENEX, USA), Bim (2819), Bad (610392, 1/250, BD transduction, USA), IKKβ (2684), IKKβ (2681), phospho-IKBα (S32/S36) (9426), NF-κB p65 (C22B4), phospho-NF-κB p65 (Ser536), 4EBP 1(9644), Phospho 4EBP-1(2855), p70S6k (2708), phospho p70S6k (9209), cyclin E (4129), cyclin A (4656) (1/1000 or 1/2000, Cell Signalling, USA), Akt (IMG-5411-1, 1/250, Imgenex, USA), phospho Akt (IMG-187-A, 1/500, Imgenex, USA)). Membranes were washed with PBS-T 5% dried milk and incubated with a secondary antibody against mice or rabbit immunoglobulin (1/3000, Caltag, USA) for 1 h. Signal was detected by chemoluminescence using ECL western blotting detection system (GE Healthcare, UK). β- actin clone AC-15 (Sigma Aldrich, St Louis, USA) served as internal control for equal loading.

### Cell cycle analysis

Cellular DNA content was determined by flow cytometry. After incubation with 10 mg/mL anti-IGF-1R and/or 10 µM AS602868LP1, RPMI8226, MM.1S and U266 cells were washed with PBS and suspended in 800 µL of propidium iodide solution (0.05 mg/mL) in the dark for 1 h at 4°C. Cells were acquired by flow cytometry and cell cycle distribution was determined using Modfit LT 2.0™ software (Veritysoftware Inc, Topsham, USA).

### Real-time quantitative RT-PCR

Total RNA was converted to cDNA using the Superscript II reverse transcriptase (Invitrogen, Cergy Pontoise, France). Real-time quantitative RT-PCR was performed using SYBR-Green technology in a Lightcycler (Roche, Mannheim, Germany) as previously described [29]. Forward and reverse primer sequences used are detailed in Table S1. Results were analyzed with RelQuant software (Roche).

### Mitochondrial membrane depolarization

Cells were incubated in the presence or absence of AS602868 or/and anti-IGF-1R antibody for 16 h, washed with phosphate-buffered saline (PBS), and incubated for 15 min at 37°C with 50 nM 3,3 dihexyloxacarbocyanine (DIOC6(3)) (Sigma Aldrich, St Louis, USA). Cells were then washed with and resuspended in PBS. DIOC6(3) fluorescence was measured by flow cytometry.

### Apoptosis assay

Cells (1.106) were cultured at 37°C with 10 µM AS602868 or/and 10 µM TPCA-1 or/and anti- IGF-1R antibody (10 µg/mL). After 48 h, cells were washed with PBS 1× and suspended in 100 µL Annexin buffer with 1 µl Annexin-V FITC (Roche, Germany). After 15 minute incubation at room temperature in the dark, cells were washed and analyzed by flow cytometry (BD Biosciences, Europe, Erembodegem, Belgium). The Annexin-V FITC positive population was considered as the apoptotic fraction in our experiments.

### Statistical analysis

Statistical comparisons were made with the Student *t*-test. The minimal level of significance was p<0.05.

## Supporting Information

Figure S1 **Cytotoxicity of anti-IGF-1R antibody in four MM cell lines.** Cells were plated in triplicate and exposed to various concentrations of anti-IGF-1R antibody and analyzed using an MTT assay.
(TIF)

Figure S2 **Cytotoxicity assays in the absence of serum.** Cells were plated in triplicate and exposed to various concentrations of AS602868 and/or anti- IGF 1R antibody 10 µg/mL.
(TIF)

Figure S3 **Western blot of IGF-1 protein.** RPMI8226 cells were incubated for 16 h with 10 µg/mL anti-IGF-1R antibody or/and 10 µM. AS602868 at 37°C.
(TIF)

Table S1 **Forward and reverse primer sequences.**
(XLSX)

Table S2 **Antibodies reference.**
(XLSX)

Table S3 **Percentage of plasma cells CD221+ and CD221−.**
(XLSX)

Table S4 **Effect of AS602868 and anti-IGF-1R on bone marrow non plasma cell compartment.**
(XLSX)

## References

[1] Kyle RA, Rajkumar SV (2004). Multiple myeloma.. N Engl J Med.

[2] Denz U, Haas PS, Wasch R, Einsele H, Engelhardt M (2006). State of the art therapy in multiple myeloma and future perspectives.. Eur J Cancer.

[3] Hsu J, Shi Y, Krajewski S, Renner S, Fisher M (2001). The AKT kinase is activated in multiple myeloma tumor cells.. Blood.

[4] Berenson JR, Ma HM, Vescio R (2001). The role of nuclear factor-kappaB in the biology and treatment of multiple myeloma.. Semin Oncol.

[5] Bharti AC, Donato N, Singh S, Aggarwal BB (2003). Curcumin (diferuloylmethane) downregulates the constitutive activation of nuclear factor-kappa B and IkappaBalpha kinase in human multiple myeloma cells, leading to suppression of proliferation and induction of apoptosis.. Blood.

[6] Giuliani N, Lunghi P, Morandi F, Colla S, Bonomini S (2004). Downmodulation of ERK protein kinase activity inhibits VEGF secretion by human myeloma cells and myeloma-induced angiogenesis.. Leukemia.

[7] Catlett-Falcone R, Landowski TH, Oshiro MM, Turkson J, Levitzki A (1999). Constitutive activation of Stat3 signalling confers resistance to apoptosis in human U266 myeloma cells.. Immunity.

[8] Ogata A, Chauhan D, Teoh G, Treon SP, Urashima M (1997). IL-6 triggers cell growth via the Ras-dependent mitogen-activated protein kinase cascade.. J Immunol.

[9] Frassanito MA, Cusmai A, Iodice G, Dammacco F (2001). Autocrine interleukin-6 production and highly malignant multiple myeloma: relation with resistance to druginduced apoptosis.. Blood.

[10] Georgii-Hemming P, Wiklund HJ, Ljunggren O, Nilsson K (1996). Insulin-like growth factor I is a growth and survival factor in human multiple myeloma cell lines.. Blood.

[11] Pene F, Claessens YE, Muller O, Viguie F, Mayeux P (2002). Role of the phosphatidylinositol 3-kinase/Akt and mTOR/P70S6-kinase pathways in the proliferation and apoptosis in multiple myeloma.. Oncogene.

[12] Mitsiades CS, Mitsiades N, Poulaki V, Schlossman R, Akiyama M (2002). Activation of NF-kappaB and upregulation of intracellular anti-apoptotic proteins via the IGF 1/Akt signalling in human multiple myeloma cells: therapeutic implications.. Oncogene.

[13] Maloney EK, McLaughlin JL, Dagdigian NE, Garrett LM, Connors KM (2003). An anti-insulin-like growth factor I receptor antibody that is a potent inhibitor of cancer cell proliferation.. Cancer Res.

[14] Martins AS, Mackintosh C, Martin DH, Campos M, Hernandez T (2006). Insulinlike growth factor I receptor pathway inhibition by ADW742, alone or in combination with imatinib, doxorubicin, or vincristine, is a novel therapeutic approach in Ewing tumor.. Clin Cancer Res.

[15] Menu E, Jernberg-Wiklund H, Stromberg T, De Raeve H, Girnita L (2006). Inhibiting the IGF-1 receptor tyrosine kinase with the cyclolignan PPP: an in vitro and in vivo study in the 5T33MM mouse model.. Blood.

[16] Cohen BD, Baker DA, Soderstrom C, Tkalcevic G, Rossi AM (2005). Combination therapy enhances the inhibition of tumor growth with the fully human anti-type 1 insulin-like growth factor receptor monoclonal antibody CP-751,871.. Clin Cancer Res.

[17] Karin M, Lin A (2002). NF-kappaB at the crossroads of life and death.. Nat Immunol.

[18] Pahl HL (1999). Activators and target genes of Rel/NF-kappaB transcription factors.. Oncogene.

[19] Regnier CH, Song HY, Gao X, Goeddel DV, Cao Z (1997). Identification and characterization of an IkappaB kinase.. Cell.

[20] Mercurio F, Murray BW, Shevchenko A, Bennett BL, Young DB (1999). IkappaB kinase (IKK)-associated protein 1, a common component of the heterogeneous IKK complex.. Mol Cell Biol.

[21] DiDonato JA, Hayakawa M, Rothwarf DM, Zandi E, Karin M (1997). A cytokineresponsive IkappaB kinase that activates the transcription factor NF-kappaB.. Nature.

[22] Hideshima T, Richardson P, Chauhan D, Palombella VJ, Elliott PJ (2001). The proteasome inhibitor PS-341 inhibits growth, induces apoptosis, and overcomes drug resistance in human multiple myeloma cells.. Cancer Res.

[23] Hideshima T, Ikeda H, Chauhan D, Okawa Y, Raje N (2009). Bortezomib induces canonical nuclear factor-kappaB activation in multiple myeloma cells.. Blood.

[24] Lounnas N, Frelin C, Gonthier N, Colosetti P, Sirvent A (2009). NF-kappaB inhibition triggers death of imatinib-sensitive and imatinib-resistant chronic myeloid leukemia cells including T315I Bcr-Abl mutants.. Int J Cancer.

[25] Sors A, Jean-Louis F, Begue E, Parmentier L, Dubertret L (2008). Inhibition of IkappaB kinase subunit 2 in cutaneous T-cell lymphoma down-regulates nuclear factor-kappaB constitutive activation, induces cell death, and potentiates the apoptotic response to antineoplastic chemotherapeutic agents.. Clin Cancer Res.

[26] Griessinger E, Frelin C, Cuburu N, Imbert V, Dageville C (2008). Preclinical targeting of NF-kappaB and FLT3 pathways in AML cells.. Leukemia.

[27] Romagnoli M, Desplanques G, Maiga S, Legouill S, Dreano M (2007). Canonical nuclear factor kappaB pathway inhibition blocks myeloma cell growth and induces apoptosis in strong synergy with TRAIL.. Clin Cancer Res.

[28] Jordheim LP, Plesa A, Dreano M, Cros-Perrial E, Keime C (2011). Sensitivity and gene expression profile of fresh human acute myeloid leukemia cells exposed ex vivo to AS602868.. Cancer Chemotherapy and Pharmacology.

[29] Jordheim LP, Cros E, Gouy MH, Galmarini CM, Peyrottes S (2004). Characterization of a gemcitabine-resistant murine leukemic cell line: reversion of in vitro resistance by a mononucleotide prodrug.. Clin Cancer Res.

[30] Sahara N, Takeshita A, Ono T, Sugimoto Y, Kobayashi M (2006). Role for interleukin-6 and insulin-like growth factor-I via PI3-K/Akt pathway in the proliferation of CD56− and CD56+ multiple myeloma cells.. Exp Hematol.

[31] Raje N, Kumar S, Hideshima T, Ishitsuka K, Chauhan D (2004). Combination of the mTOR inhibitor rapamycin and CC-5013 has synergistic activity in multiple myeloma.. Blood.

[32] Hideshima T, Chauhan D, Richardson P, Mitsiades C, Mitsiades N (2002). NFkappa B as a therapeutic target in multiple myeloma.. J Biol Chem.

[33] Birmann BM, Tamimi RM, Giovannucci E, Rosner B, Hunter DJ (2009). Insulinlike growth factor-1- and interleukin-6-related gene variation and risk of multiple myeloma.. Cancer Epidemiol Biomarkers Prev.

[34] Franchimont N, Gangji V, Durant D, Canalis E (1997). Interleukin-6 with its soluble receptor enhances the expression of insulin-like growth factor-I in osteoblasts.. Endocrinology.

[35] Guo YQ, Chen SL (2006). [The significance of IGF-1, VEGF, IL-6 in multiple myeloma progression].. Zhonghua Xue Ye Xue Za Zhi.

[36] Hideshima T, Podar K, Chauhan D, Anderson KC (2005). Cytokines and signal transduction.. Best Pract Res Clin Haematol.

[37] Jelinek DF (1999). Mechanisms of myeloma cell growth control.. Hematol Oncol Clin North Am.

[38] Sprynski AC, Hose D, Caillot L, Reme T, Shaughnessy JD (2009). The role of IGF-1 as a major growth factor for myeloma cell lines and the prognostic relevance of the expression of its receptor.. Blood.

[39] Jourdan M, De Vos J, Mechti N, Klein B (2000). Regulation of Bcl-2-family proteins in myeloma cells by three myeloma survival factors: interleukin-6, interferon-alpha and insulin-like growth factor 1.. Cell Death Differ.

[40] Jelinek DF, Witzig TE, Arendt BK (1997). A role for insulin-like growth factor in the regulation of IL-6-responsive human myeloma cell line growth.. J Immunol.

[41] Heron-Milhavet L, LeRoith D (2002). Insulin-like growth factor I induces MDM2- dependent degradation of p53 via the p38 MAPK pathway in response to DNA damage.. J Biol Chem.

[42] Qiang YW, Kopantzev E, Rudikoff S (2002). Insulinlike growth factor-I signalling in multiple myeloma: downstream elements, functional correlates, and pathway crosstalk.. Blood.

[43] Warshamana-Greene GS, Litz J, Buchdunger E, Hofmann F, Garcia-Echeverria C (2004). The insulin-like growth factor-I (IGF-I) receptor kinase inhibitor NVPADW742, in combination with STI571, delineates a spectrum of dependence of small cell lung cancer on IGF-I and stem cell factor signalling.. Mol Cancer Ther.

[44] Burtrum D, Zhu Z, Lu D, Anderson DM, Prewett M (2003). A fully human monoclonal antibody to the insulin-like growth factor I receptor blocks liganddependent signalling and inhibits human tumor growth in vivo.. Cancer Res.

[45] Bohula EA, Playford MP, Macaulay VM (2003). Targeting the type 1 insulin-like growth factor receptor as anti-cancer treatment.. Anticancer Drugs.

[46] Blum G, Gazit A, Levitzki A (2003). Development of new insulin-like growth factor-1 receptor kinase inhibitors using catechol mimics.. J Biol Chem.

[47] Warshamana-Greene GS, Litz J, Buchdunger E, Garcia-Echeverria C, Hofmann F (2005). The insulin-like growth factor-I receptor kinase inhibitor, NVP-ADW742, sensitizes small cell lung cancer cell lines to the effects of chemotherapy.. Clin Cancer Res.

[48] Ni H, Ergin M, Huang Q, Qin JZ, Amin HM (2001). Analysis of expression of nuclear factor kappa B (NF-kappa B) in multiple myeloma: downregulation of NFkappa B induces apoptosis.. Br J Haematol.

[49] Chauhan D, Hideshima T, Mitsiades C, Richardson P, Anderson KC (2005). Proteasome inhibitor therapy in multiple myeloma.. Mol Cancer Ther.

[50] Sunwoo JB, Chen Z, Dong G, Yeh N, Crowl Bancroft C (2001). Novel proteasome inhibitor PS-341 inhibits activation of nuclear factor-kappa B, cell survival, tumor growth, and angiogenesis in squamous cell carcinoma.. Clin Cancer Res.

