# Supplementary figures and images for: Inhibition of IGF-1 Signalling Enhances the Apoptotic Effect of AS602868, an IKK2 Inhibitor, in Multiple Myeloma Cell Lines
Source: PLoS One. 2011 Jul 25;6(7):e22641. doi: 10.1371/journal.pone.0022641 (PMC3143180; doi:10.1371/journal.pone.0022641)

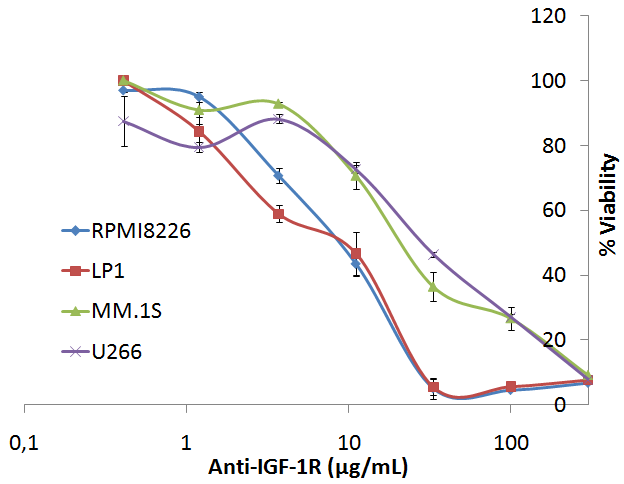

Supplement: Figure S1 — Cytotoxicity of anti-IGF-1R antibody in four MM cell lines. Cells were plated in triplicate and exposed to various concentrations of anti-IGF-1R antibody and analyzed using an MTT assay. (TIF) [file pone.0022641.s001.tif]

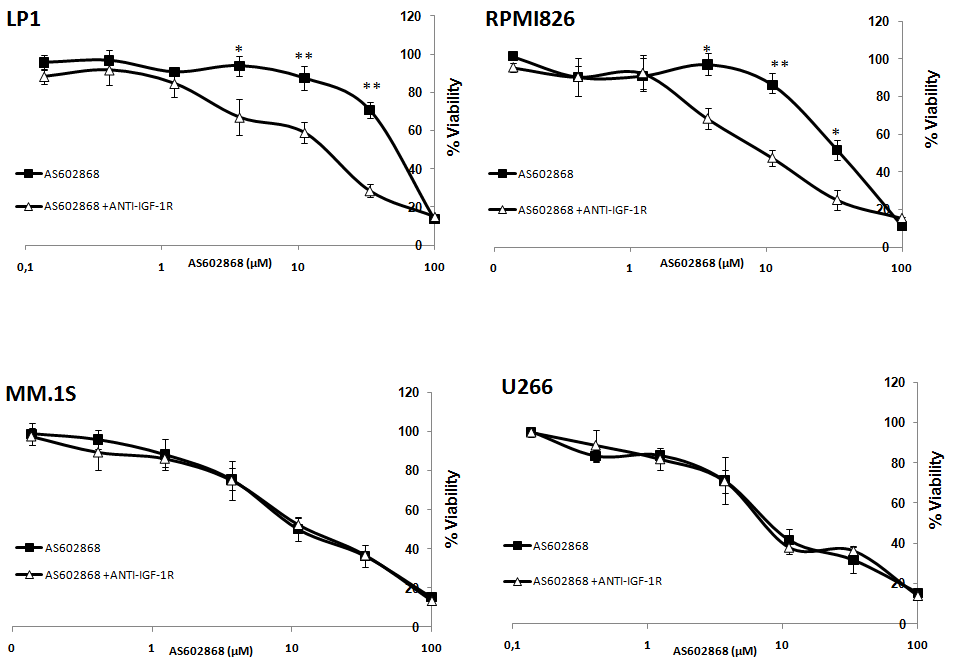

Supplement: Figure S2 — Cytotoxicity assays in the absence of serum. Cells were plated in triplicate and exposed to various concentrations of AS602868 and/or anti- IGF 1R antibody 10 µg/mL. (TIF) [file pone.0022641.s002.tif]

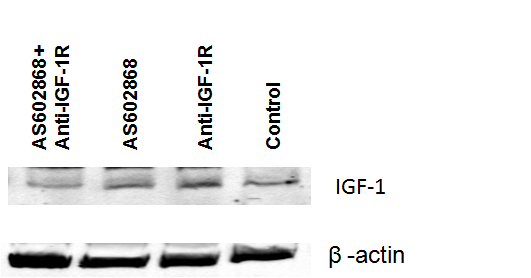

Supplement: Figure S3 — Western blot of IGF-1 protein. RPMI8226 cells were incubated for 16 h with 10 µg/mL anti-IGF-1R antibody or/and 10 µM. AS602868 at 37°C. (TIF) [file pone.0022641.s003.tif]
